# The Pharmacokinetics of the CYP3A Substrate Midazolam in Morbidly Obese Patients Before and One Year After Bariatric Surgery

**DOI:** 10.1007/s11095-015-1752-9

**Published:** 2015-07-23

**Authors:** Margreke J. Brill, Anne van Rongen, Eric P. van Dongen, Bert van Ramshorst, Eric J. Hazebroek, Adam S. Darwich, Amin Rostami-Hodjegan, Catherijne A. Knibbe

**Affiliations:** Division of Pharmacology, Leiden Academic Centre for Drug Research, Leiden University, Leiden, The Netherlands; Department of Clinical Pharmacy, St. Antonius Hospital, Koekoekslaan 1, 3435 CM Nieuwegein, The Netherlands; Department of Anaesthesiology and Intensive Care, St. Antonius Hospital, Nieuwegein, The Netherlands; Department of Surgery, St. Antonius Hospital, Nieuwegein, The Netherlands; Manchester Pharmacy School, University of Manchester, Manchester, Great Britain UK

**Keywords:** bariatric surgery, midazolam, pharmacokinetics, CYP3A

## Abstract

**Purpose:**

Bariatric surgery is nowadays commonly applied as treatment for morbid obesity (BMI > 40 kg/m^2^). As information about the effects of this procedure on a drug’s pharmacokinetics is limited, we aimed to evaluate the pharmacokinetics of CYP3A probe substrate midazolam after oral and intravenous administration in a cohort of morbidly obese patients that was studied before and 1 year post bariatric surgery.

**Methods:**

Twenty morbidly obese patients (aged 26–58 years) undergoing bariatric surgery participated in the study of which 18 patients returned 1 year after surgery. At both occasions, patients received 7.5 mg oral and 5 mg intravenous midazolam separated by 160 ± 48 min. Per patient and occasion, a mean of 22 blood samples were collected. Midazolam concentrations were analyzed using population pharmacokinetic modeling.

**Results:**

One year after bariatric surgery, systemic clearance of midazolam was higher [0.65 (7%) *versus* 0.39 (11%) L/min, mean ± RSE (*P* < 0.01), respectively] and mean oral transit time (MTT) was faster [23 (20%) *versus* 51 (15%) minutes (*P* < 0.01)], while oral bioavailability was unchanged (0.54 (9%)). Central and peripheral volumes of distribution were overall lower (*P* < 0.05).

**Conclusions:**

In this cohort study in morbidly obese patients, systemic clearance was 1.7 times higher 1 year after bariatric surgery, which may potentially result from an increase in hepatic CYP3A activity per unit of liver weight. Although MTT was found to be faster, oral bioavailability remained unchanged, which considering the increased systemic clearance implies an increase in the fraction escaping intestinal first pass metabolism.

**Electronic supplementary material:**

The online version of this article (doi:10.1007/s11095-015-1752-9) contains supplementary material, which is available to authorized users.

## Introduction

The prevalence of morbid obesity (body mass index, BMI >40 kg/m^2^) is increasing worldwide. In the United States, 6% of the population is morbidly obese while in Europe prevalence of obesity (BMI >30 kg/m^2^) ranges between 10 and 30% depending on the country (1,2). Exact numbers on the prevalence of morbid obesity in Europe are lacking, but are estimated to range between 1 and 7% (3–5).

Bariatric surgery is considered the most effective treatment for morbid obesity (6,7). In 2011, more than 340,000 bariatric surgeries, including Roux and – Y gastric bypass (RYGB) and sleeve gastrectomy, were performed worldwide (8). During a bariatric procedure, the stomach is reduced to a sleeve like structure or a small pouch and, in case of a roux and –Y gastric bypass, the duodenum and initial part of the small intestine are bypassed (9–11). These alterations in the gastro-intestinal tract may cause an increase in stomach pH, an increase in gastric emptying time, and a decrease in the surface area of absorption (9,12,13).

As such, a bariatric procedure may impact a drug’s pharmacokinetics and have consequences for dosing. In particular for drugs undergoing CYP3A metabolism, it seems relevant to study the impact of bariatric surgery, as the CYP3A enzyme resides not only in the liver but also in the gut wall and is an important drug metabolising enzyme involved in the metabolism of approximately 25% of all clinically used drugs (14). Besides bariatric surgery induced anatomical changes to the gastro-intestinal tract, the resulting reduction in (over)weight may also influence CYP3A activity itself (15). It is well known that obese patients suffer from low-grade inflammation caused by macrophages and adipocytes in the adipose tissue which excrete inflammation markers and adipokines, including Il-6 and TNF-alpha (16–18) which may lead to reduced CYP3A activity (15,19,20). As studies in morbidly obese patients before and after bariatric surgery show a reduction in inflammation status in patients after bariatric surgery, it is hypothesized that CYP3A activity in patients after bariatric surgery recovers (21,22). However, it seems that the inflammation status does not completely change back to non-obese (never been obese) individuals, as 6 months post surgery values of leptin, adiponectin and C-reactive protein (CRP) did not return to values found for lean (never been obese) patients (21).

Midazolam is considered a model substrate drug for CYP3A activity as it is primarily metabolized by CYP3A (23). Therefore, in this study we aimed to evaluate the pharmacokinetics of midazolam after oral and intravenous administration in a cohort of morbidly obese patients that was studied before and 1 year post bariatric surgery. The results are used to evaluate consequences for dosing of midazolam in patients after bariatric surgery.

## Materials and Methods

### Study Design and Patients

This is a prospective observational and interventional study in morbidly obese adult patients (NTC01519726, EudraCT 2011-003293-93). Before participation, all patients gave written informed consent. The study was approved by the local human research and ethics committee (VCMO, NL35861.100.11) and was conducted according to the principles of the Declaration of Helsinki (version 22-10-2008) and in accordance with the Medical Research Involving Human Subjects Act (WMO) of the Netherlands.

Morbidly obese patients undergoing a laparoscopic gastric bypass or sleeve surgery between 18 and 65 years were eligible for inclusion in the study. Patients were excluded if they used CYP3A inducing or inhibiting medication (24), used products containing grapefruit, wild grape, banpeiyu, pomegranate, star fruit or black berry within 2 weeks before the study, were pregnant, were breastfeeding, were younger than 18 or older than 60 years or suffered from renal insufficiency (eGFR MDRD4 < 60 mL/min).

### Study Procedures

The study consisted of two occasions. The first occasion was on the day of laparoscopic bariatric surgery (occasion 1), of which the details and results have been described in a previous report (25). One year after bariatric surgery, the 20 patients who participated on occasion 1, were invited to participate in the second part of the study (occasion 2). The period of 1 year was chosen based on the Swedish Obese Subjects study showing a mean weight loss optimum of 32% of body weight 0.5–2 years after bariatric surgery (26).

For both occasions, patients fasted from midnight until the study started in the morning (typically at 09:00 o’clock) and were not allowed to eat or drink until 1 h post intravenous midazolam dose. At first a 7.5 mg midazolam tablet was administered orally and after 160 ± 48 min an i.v. dose of 5 mg was administered. For the first occasion, the i.v. dose coincided with the induction of anesthesia for the bariatric surgical procedure while for the second occasion the i.v. dose was administered at 150 min after oral dose. Blood samples were collected at 5, 15, 30, 45, 55, 65, 75, 90, 120, 150 after oral dose and at 5, 15, 30, 60, 90, 120, 150, 180, 240 and 300, 390 and 510 min after intravenous dose at occasion 1, and at 5, 15, 30, 60, 90, 120, 150, 180, 240 and 300 min after intravenous dose at occasion 2. After collection, blood samples were centrifuged and plasma was stored at −80°C until analysis. Samples were analyzed as described before (25). The Richmond Agitation Sedation Scale (RASS) was used to score the level of sedation in each participant after midazolam oral dose until administration of the intravenous dose (27).

### Population Pharmacokinetic Analysis and Internal Validation

The population pharmacokinetic analyses was performed by means of nonlinear mixed effects modelling using NONMEM (version 7.2) (28). Pirana (2.7.1) and R (2.15) were used to visualize the data. Discrimination between different structural and statistical models was made by comparison of the objective function value (OFV, i.e., −2 log likelihood [-2LL]). A *p*-value below 0.05, representing a decrease of 3.84 in the OFV, was considered statistically significant. In addition, goodness-of-fit plots (observed *versus* individual-predicted concentrations, observed *versus* population-predicted concentrations, conditional weighted residuals *versus* time and *versus* population-predicted concentrations plots) were used for diagnostic purposes. Furthermore, the confidence interval of the parameter estimates, the correlation matrix and visual improvement of the individual plots were used to evaluate the model. The internal validity of the population pharmacokinetic model was assessed by the bootstrap re-sampling method using 500 replicates and normalized prediction distribution errors (NPDE) (29). Parameters obtained with the bootstrap replicates were compared with the estimates obtained from the original dataset and NPDE plots were checked for normal distribution characteristics and trends in the errors.

Midazolam concentration-time profiles were analysed separately (occasion 1, occasion 2) and simultaneously (occasion 1 and 2). The separate pharmacokinetic analyses allowed for initial exploration of the data and evaluation of covariate relationships within each population. For all analyses, two- and three compartment pharmacokinetics models were tested. For the description of the oral absorption phase, different models were tested including first order absorption, zero order absorption and a transit compartment model in which transit compartment rates (Ktr) were equalized to the absorption rate constant (Ka) (30). The mean oral transit time (MTT), which represents the average time for the drug from oral dose administration to appearance at the sample site, can be calculated from Ktr using MTT = (N+1)/Ktr in which N is the number of transit compartments. For the statistical model, the individual parameter estimate (Empirical Bayes Estimate or post hoc value) of the ith individual was modelled according to (Eq. 1):

1 $$ {\theta}_i={\theta}_{mean}\times \exp \left({\eta}_i\right) $$

where θ_mean_ is the population mean parameter value, and η_i_ is a random variable for the ith individual with a mean of zero and variance of ω^2^, assuming log-normal distribution in the population. The residual variability, resulting from assay errors, model misspecifications and other unexplained sources, was best described with a proportional error model. The jth observed midazolam concentration of the ith patient (Y_ij_) in Eq. 2:

2 $$ {Y}_{ij}={C}_{pred,ij}\times \left(1+{\varepsilon}_{ij}\right) $$

where C_pred_,_ij_ is the population predicted midazolam concentration of the ith individual at the jth time, and ε_ij_ is a random variable with a mean of zero and variance of σ^2^.

### Covariate Analysis

Covariates were plotted independently against the individual estimates of pharmacokinetic parameters to visualize potential relations. The following covariates were tested: body weight, BMI, lean body weight (31), age, sex and bariatric surgery. The influence of the binary covariates bariatric surgery and sex was explored by means of estimating two separate thetas or by a factor (‘Z’) of increase/decrease according to Eq. 3:

3 $$ {P}_i={P}_p\times {Z}^{COVARIATE} $$

where P_i_ and P_p_ represent the individual and population parameter estimate, Z represents the factor for increase or decrease for the patients subgroup with a Z of 1 in case the covariate equals 0 or a Z of Z in case the covariate equals 1. In case the binary covariate bariatric surgery for a specific parameter improved the model significantly, it was evaluated whether this factor of increase or decrease could be related to the difference in body weight, lean body weight (31) or BMI between occasion 1 and 2 using the following equations:

4 $$ \mathrm{If}\kern0.5em \mathrm{Occasion}=1:\ {P}_i={P}_p $$

5 $$ \mathrm{If}\kern0.5em \mathrm{Occasion}=2:{P}_i={P}_p\times factor\cdot \left(CO{V}_{BeforeSurgery}-CO{V}_{AfterSurgery}\right) $$

Furthermore, it was tested whether body weight, lean body weight, age or BMI was a linear (Eq. 7) or nonlinear (Eq. 8) covariate within occasion 1 or 2 using the following equations:

6 $$ \mathrm{If}\kern0.5em \mathrm{Occasion}=1:{P}_i={P}_p $$

7 $$ \mathrm{If}\kern0.5em \mathrm{Occasion}=2:{P}_i={P}_p\times \left(1+W\times \left(COV-CO{V}_{median}\right)\right. $$

And/or:

8 $$ \mathrm{If}\kern0.5em \mathrm{Occasion}=2:{P}_i={P}_p\times {\left(\frac{COV}{CO{V}_{median}}\right)}^X $$

where P_i_ and P_p_ represent individual and population parameter estimates, respectively; COV represents the covariate; COV_median_ represents the median covariate value; X represents the exponential scaling factor; and W represents the correlation factor between the population pharmacokinetic parameters and the covariate. The occasion conditions were switched *vice versa* to test covariate relationships within both groups.

Continuous covariates for both occasion 1 and 2 simultaneously were tested using linear and non-linear equations (Eqs. 9 and 10).

9 $$ {P}_i={P}_p\times {\left(\frac{COV}{CO{V}_{median}}\right)}^X $$

10 $$ {P}_i={P}_p\times \left(1+W\times \left(COV-CO{V}_{median}\right)\right. $$

Potential covariates were separately entered into the model and statistically tested by use of the OFV and, if applicable, the 95% CI of the additional parameter. In addition, if applicable, it was evaluated whether the interindividual variability in the parameter concerned reduced in value upon inclusion of the covariate on the parameter. After forward inclusion (*p* < 0.05), a backward exclusion procedure was applied to justify the inclusion of a covariate (*p* < 0.001). The choice of the covariate model was further evaluated as discussed above (see Population pharmacokinetic analysis and internal validation section).

### Model Simulations

The final population pharmacokinetic model was used to simulate the midazolam concentration time curves after a 7.5 mg oral dose, a 5 mg intravenous dose and a 2.5 mg/h continuous infusion. Using Monte Carlo simulations, 1000 individuals were randomly generated based on body weight distribution of our study (144 ± 26 kg) and simulations based on theta and eta values of the final PK model were performed using NONMEM.

## Results

### Patients and Data

**Fig. 1:**
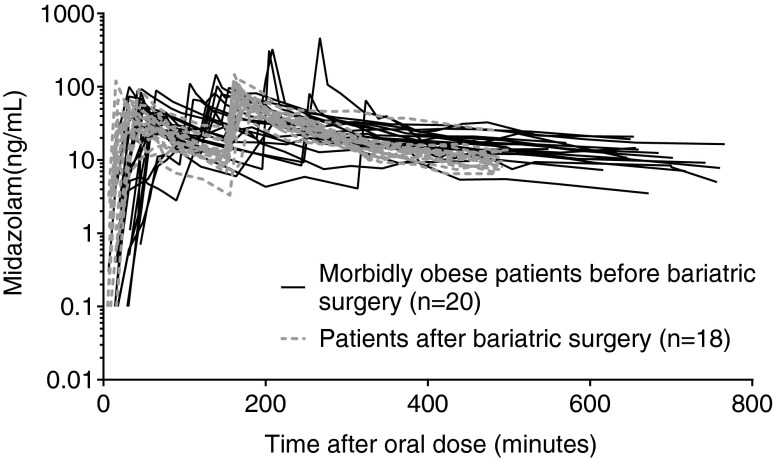
Midazolam concentration *versus* time after oral dose profiles upon a 7.5 mg oral midazolam dose and a 5 mg intravenous dose separated by 160 ± 48 min in 20 morbidly obese patients before (*black lines*) and 1 year after surgery (*grey dotted lines*). Two patients were unable to participate 1 year after surgery.

Of the 20 morbidly obese patients who participated in the first part of the trial (occasion 1), 18 patients returned 52 ± 3 weeks after bariatric surgery (occasion 2) and lost a mean of 44.5 ± 10.2 kg of body weight. Two of the 20 patients were lost to follow up to participate at occasion 2. Patients and study characteristics are summarized in Table I. Figure 1 shows the midazolam concentration time values measured at both study occasions. At occasion 1, the occurrence of the peak concentrations after the i.v. dose were found to vary largely, which resulted from differences in time of administration of the intravenous midazolam. For post-bariatric surgery patients, the concentration time curves show a slightly earlier maximum concentration (C_max_) after oral dose in comparison to morbidly obese patients before bariatric surgery, while in a few individuals of the morbidly obese patient group peak concentrations after the intravenous dose seemed higher.

**Table I:** Patients and Study Characteristics (Mean ± Standard Deviation)

	Morbidly obese patients before surgery (*n* = 20)	Minimum-maximum	Patients after bariatric surgery (*n* = 18 of 20)	Minimum-maximum
Female/Male	12/8		11/7	
Age (years)	43.6 ± 7.6	26–57	45.5 ± 7.4	27–58
Body weight (kg)	144.4 ± 21.7	112–186	98.3 ± 18.0	62–138
LBW (kg)	71.5 ± 11.9	53–95	59.5 ± 10.0	39–73
BMI (kg/m^2^)	47.1 ± 6.5	40–68	31.9 ± 5.9	24–50
Weight loss (kg)	–	–	44.5 ± 10.2	21–58
Number of midazolam samples per patient	22 ± 3	13–24	21 ± 1	19–22
Gastric bypass/ sleeve gastrectomy	–	–	16/2	–
Time post surgery (weeks)	–	–	51.8 ± 2.5	49–57

*BMI* body mass index, *LBW* lean body weight

### Pharmacokinetic Analysis

For the population pharmacokinetic analysis including the data of both occasions a three compartment model, in which the second peripheral volume was a fraction of the first peripheral compartment best described the data. Midazolam oral absorption was best described using five transit compartments, while the addition of more transit compartments did not further improve the fit of midazolam concentrations after both oral and intravenous administration. Table II shows the parameters estimates of the simple pharmacokinetic model without covariates.

**Table II:** Parameter Values of the Simple (Without Covariates) and Final (With Covariates) Population Pharmacokinetic Models and Results of the Bootstrap Analysis

Parameter	Simple model of simultaneous analysis	Final model of simultaneous analysis	Bootstrap of final simultaneous model
	Value (RSE)	Value (RSE)	Median (*2.5–97.5 percentile*)
Fixed effects			
Ka = Ktr	0.199 (11%)	–	–
Ka = Ktr_Morbidly obese_ (min^−1^)	–	0.117 (15%)	0.114 (0.08–0.15)
Ka = Ktr_Bariatric patients_ (min^−1^)	–	0.267 (19%)	0.263 (0.08–0.45)
F	0.560 (10%)	0.537 (9%)	0.543 (0.44–0.63)
CL (L/min)	0.381 (26%)	–	–
CL_Morbidly obese_ (L/min)	–	0.385 (11%)	0.366 (0.29–0.48)
fCL_Bariatric patients_(L/min)	–	1.68 (7%) (* CL_*morbidly obese*_ *= 0.647*)	1.70 (1.18–2.18) (* CL_*morbidly obese*_ *= 0.634*)
Q (L/min)	0.888 (21%)	–	–
Q_Morbidly obese_ (L/min)	–	0.669 (24%)	0.764 (0.11–1.23)
fQ_Bariatric patients_ (L/min)	–	3.22 (32%) (* Q_*Morbidly obese*_ *= 2.15*)	2.907 (−22.6–29.0) (*Q_*Morbidly obese*_ *= 3.71*)
Q2	0.644 (21%)	0.551(23%)	0.548 (0.25–0.86)
V_central_ (L)	54.7 (17%)	–	–
V_central Morbidly obese_ = V_median BW_ *(1 + X*(BW-median BW))			
V_median BW_	–	37.3 (18%)	37.2 (17.8–56.8)
X	–	0.0435 (92%)	0.052 (−0.42–0.51)
V_central Bariatric patients_ (L)	–	37.3 (18%)	37.2 ()
V_1st peripheral_ (L)	247 (30%)	–	–
V_Peripheral Morbidly obese_ = V_median BW_*(BW/median BW)^Y^			
V_median BW_	–	106 (17%)	113 (20.9–190.3)
Y	–	3.93 (20%)	3.99 (1.9–5.9)
V_1st peripheral_ _Bariatric patients_ (L)	–	106 (17%)	113 (1.9–5.9)
fV_2nd peripheral_	0.169 (25%) (*V_*1st peripheral*_ *= 42 L*)	0.359 (27%) (*V_*1st peripheral*_ *= 38 L*)	0.311 (0.13–0.58) (*V_*1st peripheral*_ *= 40 L*)
Interindividual variability (%)			
Ktr = Ka	50 (17%)	42.4 (15%)	40.6 (27–54)
CL	41.5 (24%)	19.7 (38%)	17.7 (−14–32)
F	28.6 (23%)	33.4 (18%)	32.6 (16–45)
V_central_	60.8 (20%)	53.7 (39%)	54.3 (−49–102)
V_1st peripheral_	0 FIX	0 FIX	0 FIX
Proportional residual error (%)			
	46.2 (6%)	42.1 (5%)	41.0 (12.2)
OFV	6218	5885	5997 (804)

*BW* body weight (median = 127 kg for all data), *CL* Clearance (L/min), *F* Oral bioavailability, *fCL*_*Bariatric patients*_ *(L/min)* fraction of midazolam clearance of morbidly obese patients to estimate bariatic patient clearance, *fQ*_*Bariatric patients*_ fraction of intercompartmental clearance of morbidly obese patients to estimate intercompartmental clearance of bariatric patients, *fV*_*peripheral*_ fraction of first peripheral volume of distribution to estimate second peripheral volume, *Ktr* transit compartment rate (min^−1^), *Ka* oral absorption rate (min^−1^), *OFV* Objective function value (-2LL), *Q* intercompartmental clearance (L/min), *RSE(%)* relative standard error, *V* Volume of distribution (L), *V*_*Peripheral*_ first peripheral volume of distribution

**Fig. 2:**
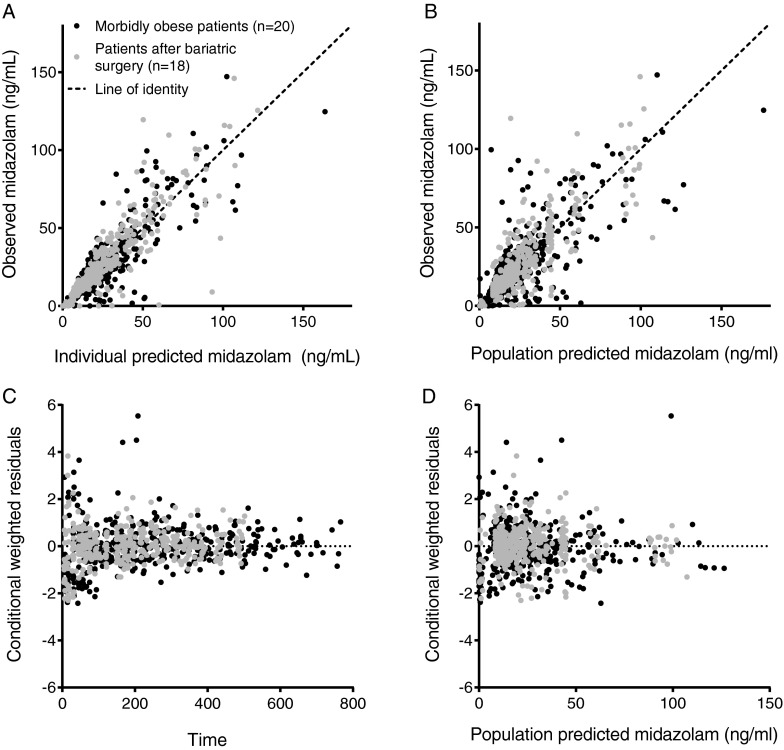
Observed *versus* individual predicted midazolam concentrations (**a**), observed *versus* population predicted midazolam concentrations (**b**), conditional weighted residuals (CWRES) *versus* time (**c**) and population predicted midazolam concentrations (**d**) of the final model for 20 morbidly obese patients (*black dots*, occasion 1) of which 18 returned 1 year post surgery for a second study visit (*grey dots*, occasion 2). The dashed line represents the line of identity (x = y).

In the covariate analysis, the binary covariate ‘bariatric surgery’ proved an important covariate for clearance (CL), oral absorption rate (Ka), inter compartmental clearance (Q) and volumes of distribution (V). For clearance, the covariate bariatric surgery gave the largest drop in OFV (−91 ΔOFV, *p* < 0.001), while a linear covariate relation with body weight resulted in a drop in OFV (−80 ΔOFV). After bariatric surgery, clearance was 1.68 times higher than in morbidly obese patients before surgery, while the extent of this increase could not be related to the loss in (lean) body weight (*p* > 0.05, Table II). Bariatric surgery as covariate on Ka also resulted in an improved fit of the model (−167 ΔOFV, *p* < 0.001). In the final model, Ka was separately estimated for both occasions and was found to have a larger value in patients after bariatric surgery (0.117 *versus* 0.267 min^−1^, Table II). As a consequence, the mean oral transit time (MTT), which is calculated from the oral absorption rate, was 51.3 (15%) before *versus* 22.6 (19%) minutes after bariatric surgery. Furthermore, bariatric surgery resulted in a 3.22 times increase in inter compartmental clearance, Q (0.669 to 2.15 L/min, −16 ΔOFV, *p* < 0.001). Finally, central and the peripheral volumes of distribution were overall lower in patients after bariatric surgery without a significant influence of (lean) bodyweight within this group. In the morbidly obese patients group, central and peripheral volume increased with body weight (−6 ΔOFV, *p* < 0.02 and −68 ΔOFV, *p* < 0.001, respectively, Table II). As the second peripheral volume of distribution was modeled as a fraction of the first peripheral volume of distribution, for morbidly obese patients before surgery it varied with body weight in a similar manner as the first peripheral volume of distribution (Table II). Concerning oral bioavailability (F) and inter-compartmental clearance to the second peripheral compartment (Q2) none of the covariates were of significant influence (*p* > 0.05). Parameters estimates of the final pharmacokinetic model are shown in Table II and goodness of fit plots are shown in Fig. 2. A 500 replicate bootstrap showed validity of the model (94% successful, Table II) and NPDE plots are presented in electronic supplementary material and showed a normal distribution of errors without any trends for both occasions.

**Fig. 3:**
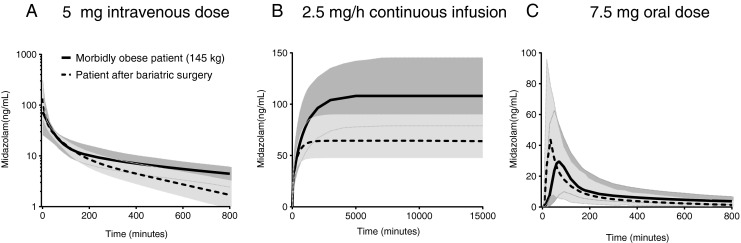
Population mean (*black line*) and 90% interval (*grey area*) of midazolam concentrations *versus* time after a 5 mg intravenous dose (**a**), a 2.5 mg/h continuous infusion (**b**) and a 7.5 mg oral dose (**c**) in morbidly obese patients before bariatric surgery (*black solid line*) and after bariatric surgery (*black dotted line*).

In Fig. 3 the population mean and 90% confidence interval of 1000 Monte Carlo midazolam dose simulations for morbidly obese patients before and after surgery are presented. After a 5 mg intravenous dose, midazolam concentrations in a bariatric surgery patient show a higher initial midazolam concentration and a faster decline over time compared to a morbidly obese patient before surgery (Fig. 3a). Upon a midazolam 2.5 mg/h continuous infusion a bariatric patient is exposed to a lower steady state concentration in comparison to a morbidly obese patient (Fig. 3b), while steady state concentrations are reached approximately 2.5 times faster in bariatric patients (~14 h) than in morbidly obese patient (~37 h). Finally, oral midazolam in a bariatric patient will result in a shorter time to maximum concentration (T_max_, 32 *versus* 65 min) and 1.5 times increase in midazolam C_max_ in comparison to before surgery (Fig. 3c).

## Discussion

**Fig. 4:**
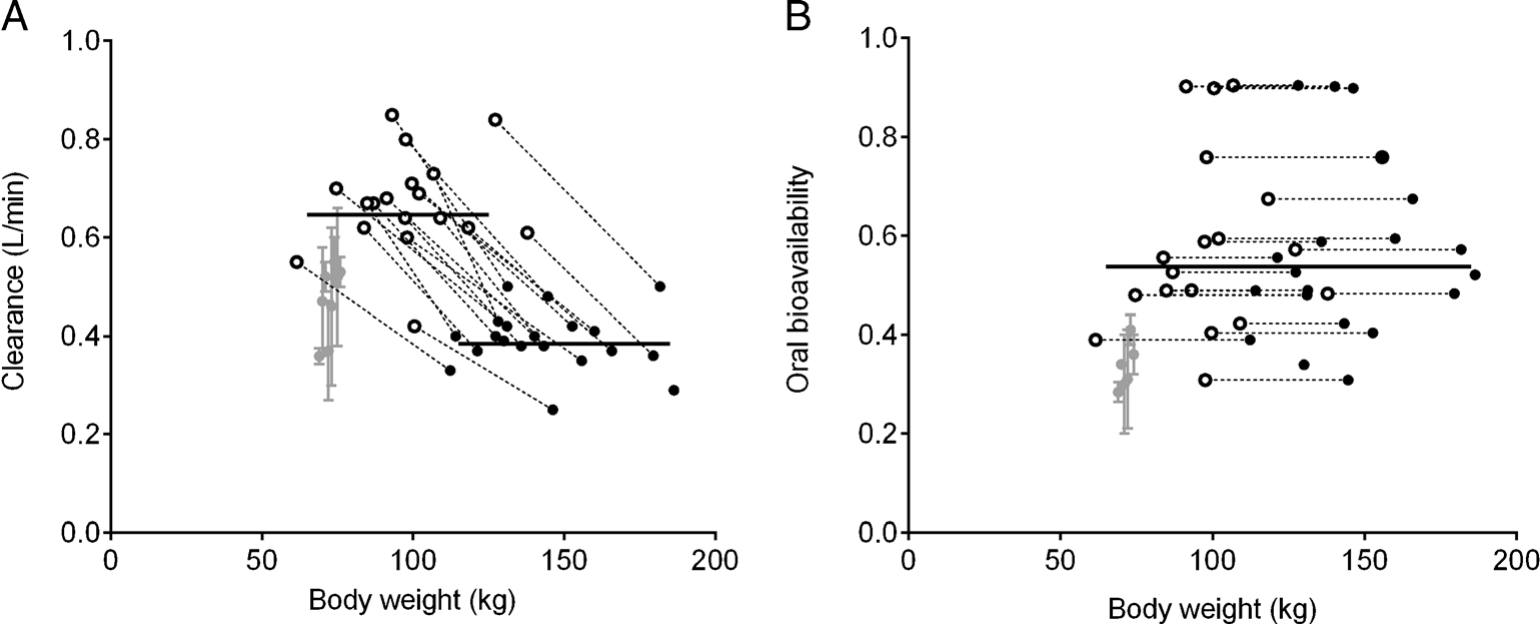
Empirical bayes estimates (*black dots*) and population mean estimates (*black lines*) of midazolam clearance (**a**) and oral bioavailability (**b**) of the final pharmacokinetic model in 20 morbidly obese patients (*black closed dots*) and 18 bariatric patients (*black open dots*) *versus* body weight (kg). Parameter values found for healthy volunteers studies from the literature were added for comparison (*grey dots*) (25,34–40).

In this cohort study in which morbidly obese patients are studied until 1 year after bariatric surgery, we aimed to determine how and to what extent midazolam pharmacokinetics after oral and intravenous administration are affected by bariatric surgery. One year post bariatric surgery, we found that midazolam systemic clearance and mean oral transit time were substantially increased while oral bioavailability remained unchanged. Central and peripheral volumes of distribution were generally lower in patients after bariatric surgery. The main finding of this study is the substantial increase in midazolam systemic clearance in all 18 patients 1 year after bariatric surgery compared to their values before surgery. This increase in clearance after bariatric surgery could not be contributed to the decrease in body weight as the body weight model was inferior to the bariatric surgery model (*p* < 0.05). (Fig. 4). Hepatic CYP3A protein expression in liver biopsies has been reported to be unaltered after bariatric surgery indicating unchanged CYP3A mediated clearance (32). However, Tandra *et al.* also found increased systemic clearance of midazolam in 18 bariatric patients >1 year post RYGB surgery in comparison with 18 controls (1.57 ± 0.95 *versus* 0.92 ± 0.72 L/min, *p* = 0.03) (33). In their study, control patients were matched for age, sex, race, and body mass index, while in our study we compared midazolam pharmacokinetics within the same cohort using a follow up design. Comparing our values to systemic clearance values in healthy volunteers (Fig. 4a), it seems that systemic clearance values post bariatric surgery are higher than those of healthy volunteers found in the literature (25,34–40). We anticipate that the increase in systemic midazolam clearance may be explained by a recovery of hepatic CYP3A activity due to decreased inflammation status, as many studies have shown a reduction in inflammatory adipokines in the plasma of patients after bariatric surgery (22). Moreover, it has been shown in *in vitro* and animal studies that a fatty liver, which is highly associated with morbid obesity, represses CYP3A activity (41,42). While CYP3A activity may have recovered 1 year after bariatric surgery, Immonen *et al.* showed on the other hand that 6 months after bariatric surgery both the fat content and size of the liver is reduced to almost the level of lean subjects, which could imply a reduced clearance (21). The fact that we identify in our study an increase in systemic midazolam clearance post bariatric surgery implies that the increased CYP3A activity per unit of liver compensates and surpasses the reduction in liver size that is associated with bariatric surgery in these patients. Another explanation could be a recovery in total liver blood flow, due to recovery of fatty liver and/or steatosis (43), but as midazolam is an intermediate extraction ratio drug this seems unlikely (44).

Midazolam mean oral transit time was twice as fast in patients 1 year post bariatric surgery in comparison to before surgery. Decreased T_max_ in bariatric surgery patients has been reported before for oral caffeine, tolbutamide, midazolam, omeprazole and duloxetine administration (33,45). The faster midazolam oral absorption may be explained by faster gastric emptying of the stomach due to the reduced stomach size (12,13,46,47). In contrast to more rapid oral absorption, for oral bioavailability, we found no difference before and after bariatric surgery. From a comparison to healthy volunteers (Fig. 4b), it can be concluded that oral bioavailability values in bariatric patients do not seem to return to values found for healthy volunteers, but remain at the level of those found for morbidly obese patients. The oral bioavailability value (F) may be deduced to its individual contributors, which are the fraction absorbed (f_a_), the fraction escaping gut wall metabolism (F_G_) and the fraction escaping first pass hepatic metabolism (F_H_).

As midazolam is a highly soluble and permeable drug, f_a_ can be assumed to be equal to 1 in morbidly obese patients before and after surgery (48). In addition, assuming no change in hepatic blood flow and blood to plasma partition ratio before and after surgery, F_H_ will decrease approximately 1.68 times post bariatric surgery as a result of 1.68 times increased systemic clearance. So, given the unchanged total bioavailability, F_total,_ we identified in our study, this implies that the midazolam fraction escaping gut wall (F_G_) increases 1.68 times 1 year after bariatric surgery. An increase in F_G_ was also predicted by Darwich *et al.*, who showed that post RYGB surgery the F_G_ of CYP3A substrate simvastatin increased with 13% (49). Increased F_G_ may be due to the bypass of the intestines resulting from this type of surgery, in which normally approximately 75% of the midazolam dose would have been absorbed (48,50). Another explanation could be an increase in splanchnic blood flow resulting in an increase in F_G_, however this seems very unlikely in view of the decrease in bodyweight associated with bariatric surgery and therefore an anticipated decrease in splanchnic blood flow instead of increase.

For midazolam central and peripheral volume of distribution we observed overall lower values in post bariatric surgery patients without variation due to body weight (*p* > 0.05, Table II). While we anticipate that this is due to the smaller range in bodyweight in the bariatric patient group, as within the morbidly obese patient group volume of distribution was highly depended on body weight as was reported before (25). To account for the influence of body weight on both the first and second peripheral volume of distribution, the second peripheral volume was modeled as a fraction of the first volume of distribution. The general reduction in volume of distribution after bariatric surgery may result from weight loss resulting in substantial reductions in blood volume and adipose tissue (51).

Although this study provides unique information on the pharmacokinetics of midazolam after both oral and intravenous dose administration in a new and emerging patient population, the study may have some limitations. First, 2 of the 18 patients underwent a sleeve gastrectomy procedure, which is an insufficient number to draw any conclusion on the effect of a sleeve gastrectomy on midazolam pharmacokinetics. However, these 2 patients did show a major loss in body weight to an extent that was similar to that of the 16 gastric bypass patients, which was the reason why we included these patients in the analysis. Moreover, when excluding these two patients from the dataset, none of parameter estimates were significantly different (data not shown). Second, at occasion 1, patients underwent surgery and anesthesia, which was not the case during occasion 2. This may potentially have influenced the results on midazolam PK we report in this study. It is well known that during a surgery cardiac output is lowered which may have caused lower midazolam clearances for morbidly obese patients. However, the duration of surgery and anesthesia was quite limited (86.4 ± 31 min) in comparison to the study period of the first occasion (~660 min), minimizing the influence of surgery. Moreover, bariatric surgery was performed using minimally invasive techniques (laparoscopic techniques) reducing hemodynamically induced changes. Furthermore, surgery was performed 159 ± 67 min after oral midazolam dose administration, which excludes any influence of surgery/anesthesia on midazolam the oral absorption phase. For these reasons, we think that the short duration of surgery/anesthesia during the first occasion is not of significant influence on the conclusion drawn based on these data.

The midazolam dose simulations provide insight in how the altered pharmacokinetics in bariatric patients affect midazolam concentration time profiles after oral or intravenous administration. A 5 mg intravenous midazolam bolus dose results in higher initial midazolam concentrations in a patient post bariatric surgery than in a morbidly obese patient. This indicates that, in comparison to morbidly obese patients, a lower intravenous bolus dose may be anticipated in patients post bariatric surgery, as after a intravenous bolus dose, midazolam effect is primarily determined by the initial concentrations. For an continuous intravenous infusion, a lower steady state concentration is reached in a bariatric patient due to the almost doubled midazolam clearance value compared to morbidly obese patients. So to reach a similar steady state concentration in a bariatric patient a higher mg/h dose seems necessary. In this respect, it is important to realise that a post-bariatric surgery patient does not only need a higher dose than before surgery but also may need a higher dose in mg/h than a non-obese patient given the differences in clearance values (Fig. 4). Furthermore, the steady state concentration is reached 2.5 times faster in a bariatric surgery patient compared to a morbidly obese patient. Finally, a midazolam oral tablet will result in increased C_max_ and earlier T_max_ in a bariatric patient.

Finally, the influence of bariatric surgery on midazolam systemic clearance found in this study may be extrapolated to other drugs which are also primarily metabolised by CYP3A, as midazolam is considered a CYP3A probe substrate (23). While the extrapolation potential depends on many factors, including extraction ratio and physico-chemical properties of the drug, it may be speculated that other major CYP3A substrates may show a similar effect of bariatric surgery on systemic clearance.

## Conclusion

In conclusion, in this cohort study in morbidly obese patients, systemic clearance was 1.7 times higher 1 year after bariatric surgery, which may potentially result from an increase in hepatic CYP3A activity per unit of liver. Even though mean oral transit time was found to be faster, oral bioavailability remained unchanged, which considering the increased systemic clearance implies an increase in the midazolam fraction escaping intestinal first pass metabolism after an oral administration. In patients after a bariatric surgery, these alterations will result in lower midazolam steady state concentrations and in higher and earlier peak concentrations after oral administration in comparison to morbidly obese patients before bariatric surgery.

## Electronic supplementary material

ESM 1 (PDF 35 kb)

## Abbreviations

MTT: Mean oral transit time
NPDE: Normalized prediction distribution errors
OFV: Objective function value
RYGB: Roux and – Y gastric bypass
