# Supplementary material for: The Pharmacokinetics of the CYP3A Substrate Midazolam in Morbidly Obese Patients Before and One Year After Bariatric Surgery
Source: Pharm Res. 2015 Jul 23;32(12):3927–36. doi: 10.1007/s11095-015-1752-9 (PMC4628089; doi:10.1007/s11095-015-1752-9)
Supplement: Supplementary file 1 — (PDF 35 kb) [file 11095_2015_1752_MOESM1_ESM.pdf]

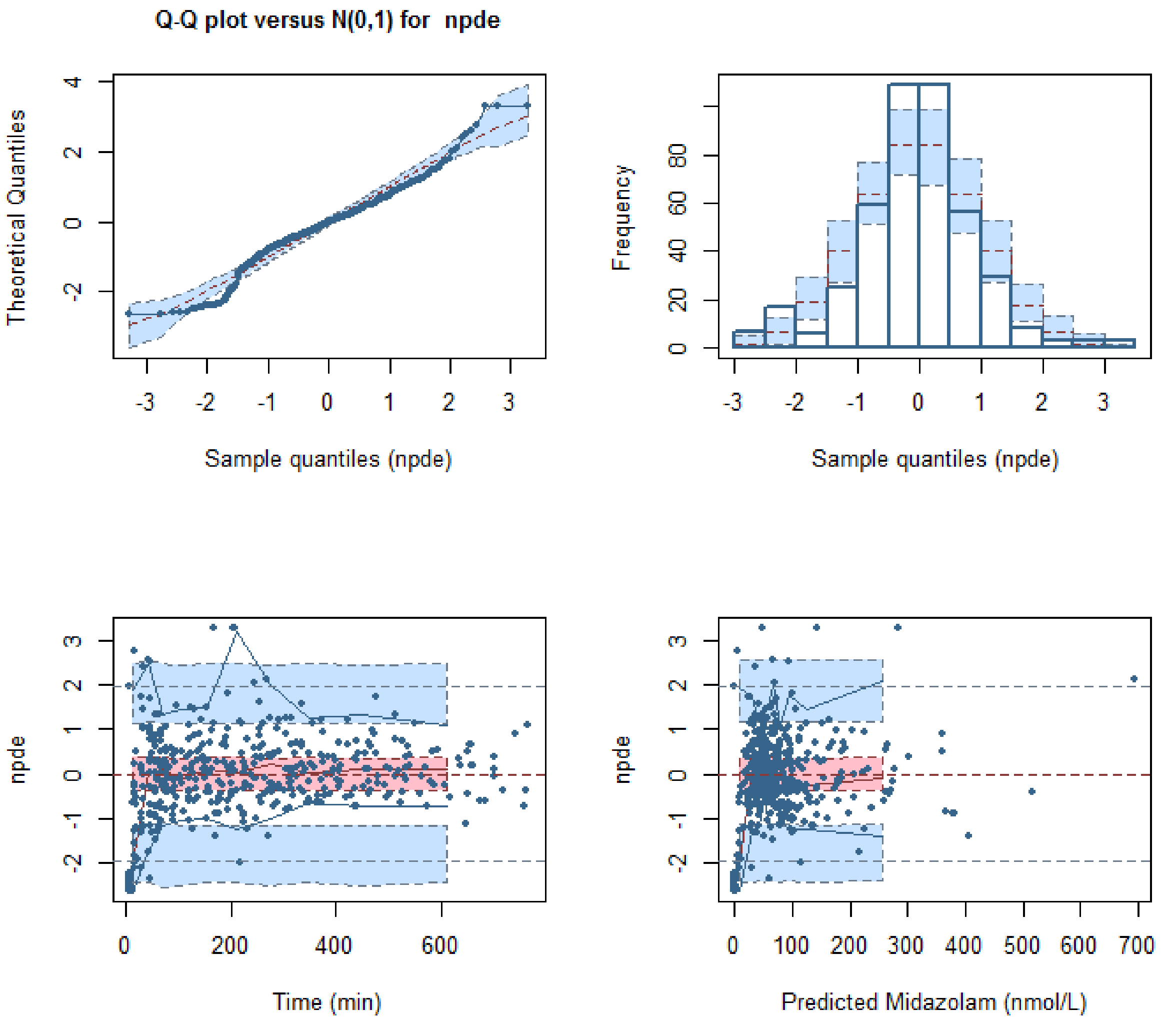

Supplemental Digital Content 1A Normalised prediction distribution errors plots of the final pharmacokinetic model of midazolam concentrations in 20 morbidly obese patients before bariatric surgery.

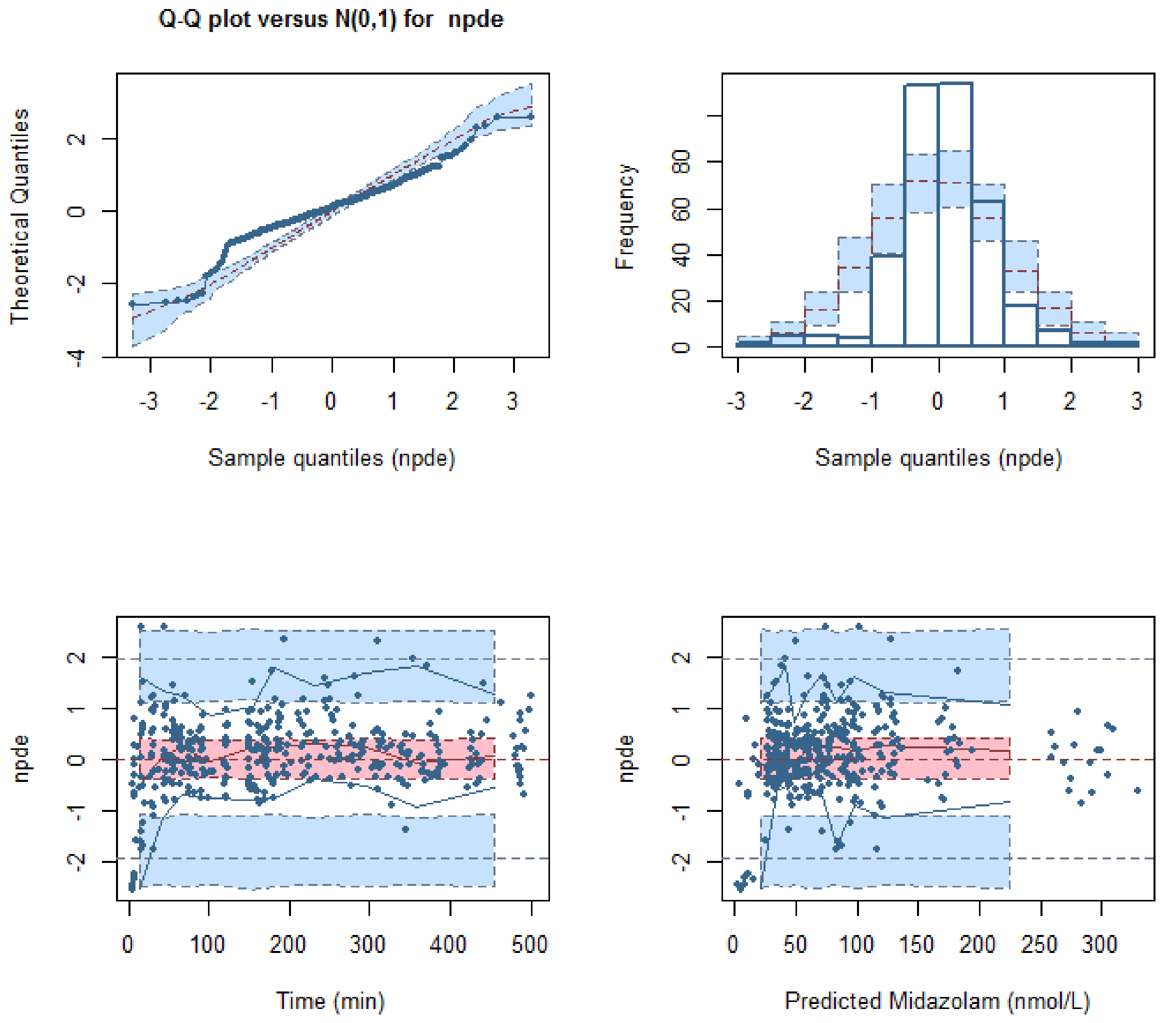

Supplemental Digital Content 1B Normalised prediction distribution errors plots of the final pharmacokinetic model of midazolam concentrations in 18 patients whom returned one year after bariatric surgery.
